# Evidence based recommendations for diagnosis and treatment of cryopyrin-associated periodic syndromes (CAPS)

**DOI:** 10.1186/1546-0096-12-S1-P78

**Published:** 2014-09-17

**Authors:** Nienke Ter Haar, Marlen Oswald, Luca Cantarini, Marco Gattorno, Michael Hofer, Isabelle Kone-Paut, Jordi Anton-Lopez, Karyl Barron, Paul Brogan, Joost Frenkel, Caroline Galeotti, Gilles Grateau, Veronique Hentgen, Tilmann Kallinich, Helen Lachmann, Huri Ozdogan, Seza Ozen, Anna Simon, Yosef Uziel, Carine Wouters, Brian Feldman, Bas Vastert, Nico Wulffraat, Susanne Benseler, Jasmin Kümmerle-Deschner

**Affiliations:** 1 Laboratory for Translational Immunology, University Medical Center Utrecht, Utrecht, Netherlands; 2 Klinik für Kinder- und Jugendmedizin, Abteilung für pädiatrische Rheumatologie , Autoinflammation Reference Center Tübingen, Universitätsklinikum Tübingen, Tübingen, Germany; 3 Policlinico Le Scotte, University of Siena, Siena, Italy; 4 G. Gaslini Institute, Genova, Italy; 5 Department of Pediatrics, University of Lausanne and, University of Geneva, Switzerland; 6 Department of Pediatric Rheumatology, Reference Centre for Autoinflammatory Disorders CEREMAI, Bicêtre Hospital, University of Paris SUD, Paris, France; 7 Hospital Sant Joan de Déu, Universitat de Barcelona, Barcelona, Spain; 8 NIH, Bethesda, USA; 9 Department of Rheumatology, UCL Institute of Child Health, London, UK; 10 Department of Pediatrics, University Medical Center Utrecht, Utrecht, Netherlands; 11 Reference Centre for Autoinflammatory Disorders CEREMAI, Bicêtre Hospital, University of Paris SUD, Paris, France; 12 Centre national de référence des amyloses d'origine inflammatoire et de la fièvre , Hôpital Tenon,AP-HP, université Pierre-et-Marie-Curie, Paris, France; 13 Centre Hospitalier de Versailles, Le Chesnay Cedex, France; 14 Charite University Medicine, Berlin, Germany; 15 National Amyloidosis Centre, University College London Medical School, London, UK; 16 Cerrahpasa Ic Hastaliklari Klinigi, Istanbul Turkey; 17 Department of Pediatrics, Hacettepe University Faculty of Medicine, Ankara, Turkey; 18 Department of Medicine, Radboudumc, Nijmegen, Netherlands; 19 Department of Pediatrics, Sapir Medical Center, Kfar Saba, Tel-Aviv University, Sackler School of Medicine, Tel-Aviv, Israel; 20 University Hospital Leuven, Leuven, Belgium; 21 Division of Rheumatology, The Hospital for Sick Children, Toronto, Canada; 22 Department of Pediatric Immunology, University Medical Center Utrecht, Utrecht, Netherlands; 23 The Hospital for Sick Children, Toronto, Canada

## Introduction

Cryopyrin-associated periodic syndromes (CAPS) is a group of rare monogenetic autoinflammatory disorders. Evidence-based guidelines are lacking and management is mostly based on physician’s experience. Consequently, treatment regimens differ throughout Europe. In 2012, a European initiative called SHARE (*Single Hub and Access point for pediatric Rheumatology in Europe*) was launched to optimize and disseminate diagnostic and management regimens in Europe for children and young adults with rheumatic diseases.

## Objectives

One of the aims of SHARE was to provide evidence based recommendations for management (treatment and monitoring) of CAPS.

## Methods

Evidence based recommendations were developed using the European League Against Rheumatism (EULAR) standard operating procedure. An expert committee was instituted, consisting of pediatric and adult rheumatologists. The expert committee defined search terms for the systematic literature review. Two independent experts scored articles for validity and level of evidence. Recommendations derived from the literature were evaluated by an online survey. Those with less than 80% agreement on the online survey or with relevant comments of the experts were reformulated. Subsequently, all recommendations were discussed at a consensus meeting using Nominal Group Technique. Recommendations were accepted if more than 80% agreement was reached.

## Results

The literature search yielded 1698 articles, of which 25 papers on treatment were considered relevant and therefore scored for validity and level of evidence. Seventeen were scored valid and used in the formulation of the recommendations. Fifteen recommendations were suggested in the online survey and discussed during the consensus meeting. Six general recommendations on management, five for monitoring and four for treatment were accepted with more than 80% agreement. Topics covered are the following: the multidisciplinary team, treatment goals, adjunctive therapies, psychosocial support and vaccinations [general recommendations], monitoring frequency, minimal assessments in all CAPS patients and monitoring of severe phenotypes [monitoring] and IL-1 blockade, NSAIDs and/or glucocorticoids during attacks and DMARDS/biologicals other than IL-1 blockade [treatment].

## Conclusion

The SHARE initiative provides recommendations for the management of CAPS and thereby facilitates improvement and uniformity of care throughout Europe.

## Disclosure of interest

N. Ter Haar Grant / Research Support from: SHARE is funded by the European Commission ( project N° 20111202), M. Oswald: None declared., L. Cantarini Grant / Research Support from: Novartis, SOBI, Consultant for: Novartis, SOBI, M. Gattorno Grant / Research Support from: Novartis, Speaker Bureau of: SOBI, M. Hofer: None declared., I. Kone-Paut Grant / Research Support from: Chugai, Novartis, SOBI, Consultant for: Abbvie, Chugai, Novartis, Pfizer, SOBI, Speaker Bureau of: Novartis, Pfizer, J. Anton-Lopez Grant / Research Support from: Abbvie, Novartis, Pfizer, Consultant for: Novartis, Speaker Bureau of: Abbvie, Novartis, Pfizer, Roche, SOBI, K. Barron: None declared., P. Brogan Grant / Research Support from: Novartis, Roche, Consultant for: Novartis, J. Frenkel Consultant for: Novartis, Speaker Bureau of: SOBI, C. Galeotti Grant / Research Support from: Novartis, G. Grateau Consultant for: Novartis, V. Hentgen Consultant for: Novartis, T. Kallinich Grant / Research Support from: Novartis, Speaker Bureau of: Novartis, SOBI, H. Lachmann: None declared., H. Ozdogan: None declared., S. Ozen Consultant for: Novartis, Speaker Bureau of: Biovitrium, A. Simon Consultant for: Novartis, SOBI, Xoma, Y. Uziel Grant / Research Support from: Novartis, Consultant for: Novartis, Speaker Bureau of: Abbvie, Neopharm, Novartis, Roche, C. Wouters: None declared., B. Feldman: None declared., B. Vastert Consultant for: Novartis, N. Wulffraat Grant / Research Support from: Abbvie, GSK, Roche, Consultant for: Genzyme, Novartis, Pfizer, Roche, S. Benseler: None declared., J. Kümmerle-Deschner Grant / Research Support from: Novartis, Speaker Bureau of: SOBI.

